# Advancements in Liquid Jet Technology and X-ray Spectroscopy for Understanding Energy Conversion Materials during Operation

**DOI:** 10.1021/acs.accounts.2c00525

**Published:** 2023-01-13

**Authors:** Torben Reuss, Sreeju Sreekantan Nair Lalithambika, Christian David, Florian Döring, Christian Jooss, Marcel Risch, Simone Techert

**Affiliations:** † Deutsches Elektronen-Synchrotron DESY, Notkestr. 85, 22607 Hamburg, Germany; ‡ Paul Scherrer Institute, Forschungsstrasse 111, 5232 Villigen-PSI, Switzerland; § Institute of Material Physics, Göttingen University, Friedrich Hund Platz 1, 37077 Göttingen, Germany; ∥ Institute for X-ray Physics, Göttingen University, Friedrich Hund Platz 1, 37077 Göttingen, Germany

## Abstract

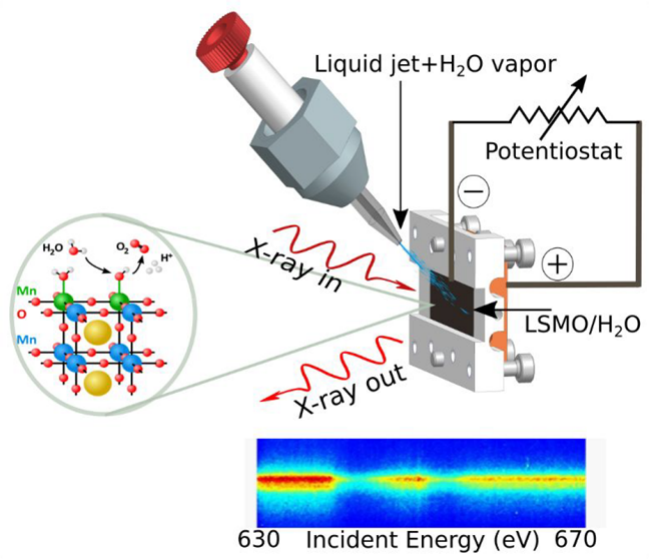

Water splitting is intensively studied for sustainable and effective energy storage in green/alternative energy harvesting–storage–release cycles. In this work, we present our recent developments for combining liquid jet microtechnology with different types of soft X-ray spectroscopy at high-flux X-ray sources, in particular developed for studying the oxygen evolution reaction (OER). We are particularly interested in the development of *in situ* photon-in/photon-out techniques, such as in situ resonant inelastic X-ray scattering (RIXS) techniques at high-repetition-frequency X-ray sources, pointing toward *operando* capabilities. The pilot catalytic systems we use are perovskites having the general structure ABO_3_ with lanthanides or group II elements at the A sites and transition metals at the B sites. Depending on the chemical substitutions of ABO_3_, their catalytic activity for OER can be tuned by varying the composition.

In this work, we present our *in situ* RIXS studies of the manganese L-edge of perovskites during OER. We have developed various X-ray spectroscopy approaches like transmission zone plate-, reflection zone plate-, and grating-based emission spectroscopy techniques. Combined with tunable incident X-ray energies, we yield complementary information about changing (inverse) X-ray absorption features of the perovskites, allowing us to deduce element- and oxidation-state-specific chemical monitoring of the catalyst. Adding liquid jet technology, we monitor element- and oxidation-state-specific interactions of the catalyst with water adsorbate during OER. By comparing the different technical spectroscopy approaches combined with high-repetition-frequency experiments at synchrotrons and free-electron lasers, we conclude that the combination of liquid jet with low-resolution zone-plate-based X-ray spectroscopy is sufficient for element- and oxidation-state-specific chemical monitoring during OER and easy to handle.

For an in-depth study of OER mechanisms, however, including the characterization of catalyst–water adsorbate in terms of their charge transfer properties and especially valence intermediates formed during OER, high-resolution spectroscopy tools based on a combination of liquid jets with gratings bear bigger potential since they allow resolution of otherwise-overlapping X-ray spectroscopy transitions. Common for all of these experimental approaches is the conclusion that without the versatile developments of liquid jets and liquid beam technologies, elaborate experiments such as high-repetition experiments at high-flux X-ray sources (like synchrotrons or free-electron lasers) would hardly be possible. Such experiments allow sample refreshment for every single X-ray shot for repetition frequencies of up to 5 MHz, so that it is possible (a) to study X-ray-radiation-sensitive samples and also (b) to utilize novel types of flux-hungry X-ray spectroscopy tools like photon-in/photon-out X-ray spectroscopy to study the OER.

## Key References

- HallmannJ.; GrübelS.; RajkovicI.; QuevedoW.; BusseG.; ScholzM.; MoreR.; PetriM.; TechertS.First steps toward probing chemical reaction dynamics with free-electron laser radiationradiation—case studies at the FLASH facility. J. Phys. B: At., Mol. Opt. Phys.2010, 43, 194009.^1^*This experimental research paper invents the liquid jet technology for free-electron lasers and demonstrates that the liquid jet can be used in X-ray scattering but also in X-ray spectroscopy setups (allowing extension to synchrotron experiments too).*
- BusseP.; YinZ.; MierwaldtD.; ScholzJ.; KressdorfB.; GlaserL.; MiedemaP.S.; RothkirchA.; ViefhausJ.; JoossC.; TechertS.; RischM.Probing the surface of La_0.6_Sr_0.4_MnO_3_ in water vapor by *in situ* photon-in/photon-out spectroscopy. J. Phys. Chem. C2020, 124, 7893–7902.^2^*This experimental research paper introduces resonant inelastic X-ray scattering at synchrotron sources for surface studies of the electronic properties of complex catalysts. The studies concentrate on perovkites without/with water vapor and confirm former X-ray absorption studies.*
- RaabeS.; MierwaldtD.; CistonJ.; UijttewaalM.; SteinH.; HoffmanJ.; ZhuY.; BlöchlP.; JoossC.In situ electrochemical electron microscopy study of oxygen evolution activity of doped manganite perovskites. Adv. Funct. Mater.2012, 22, 3378–3388.^3^*In this work, in situ electron microscopy is introduced for studying perovskite catalysts for the oxygen evolution reaction. Complementary to in situ X-ray spectroscopy, local information about the catalytic activities of the perovskite surface under in situ conditions can be derived.*

## Introduction

One of the greatest challenges of our current time lies in the systematic research and characterization of chemical reactions that allow the storage and output of energy in a highly efficient manner while conserving natural resources. In the field of the production of so-called “green hydrogen”, the hydrogen evolution reaction (HER) as well as the oxygen evolution reaction (OER), i.e., the production of hydrogen and oxygen by water splitting and its back reaction, belong to the most important and currently most studied chemical reactions of water utilization for energy storage and production.^4−13^ In the field of high-flux X-ray sources, for both synchrotrons and free-electron lasers,^14^ this means developing new measurement techniques and methods that provide an alternate and additional view on HER or OER alongside existing measurement techniques, leading to improvements in electrochemically and photoelectrochemically active catalysts and their mode of action.

In soft X-ray spectroscopy, various groundbreaking techniques have been developed for studying OER, focusing on X-ray absorption techniques *in situ*(15,16) and *operando*.^17−19^ The studies concentrate on detecting the interacting X-rays in absorption mode either by direct transmission absorption experiments or by inverse absorption techniques derived from emission spectra (as explained in the following paragraph). All in all, the X-ray spectroscopies allow monitoring of element-specific oxidation state changes during OER. Insight into hole and electron generation in relation to the band gap properties of the catalytic materials can also be obtained.

**Figure 1:**
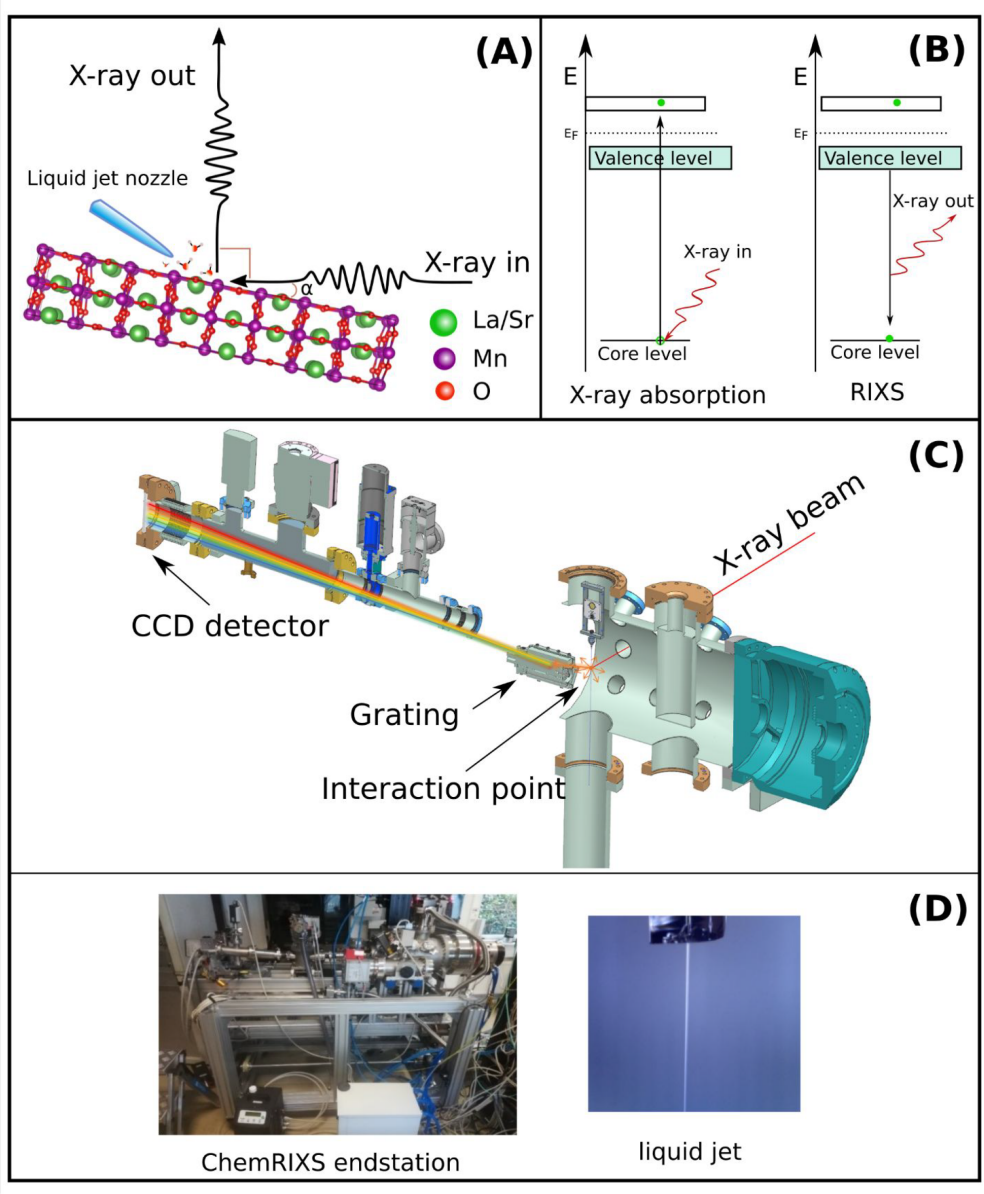
(A, B) Principle of resonant inelastic X-ray scattering (RIXS) and difference between X-ray absorption and RIXS excitation schemes. (C) The ChemRIXS chamber, designed for *in situ* and *operando* studies with ChemRIXS. (D) Photographs of the ChemRIXS chamber and the liquid jet.

With the presented resonant inelastic X-ray scattering (RIXS) experiments we want to add another precision step by determining intermediate species between the catalyst and the water on the surface of the catalyst. To follow the line of already developed techniques, over the last years we have paid particular attention to the development of time-dependent X-ray methods such as time-resolved and ultrafast X-ray diffraction or time-resolved multidimensional soft X-ray spectroscopy for studying chemical reactions beyond X-ray absorption spectroscopy.^1^ The development of flux-hungry X-ray spectroscopy techniques to high resolution (Figure 1A) has become possible since we were technically able to couple the liquid jet with its megahertz sample exchange repetition frequency with different photon-in/photon-out X-ray spectrometers. RIXS^11,12^ is a photon-in/photon-out spectroscopy technique where the outgoing energy of photons scattered by the sample is detected as a function of the incident X-ray energy. Figure 1A shows such a photon-in/photon-out scheme on a perovskite sample, one of our pilot systems. Essentially, RIXS is X-ray emission spectroscopy with tuning of the incident X-ray energy. On an atomistic level, the differences between X-ray absorption spectroscopy (XAS) and RIXS are shown in Figure 1B. Staying with the perovskite example, with soft X-ray spectroscopy, the shown generalized transition diagram applies for the transitions and levels involved in RIXS of the oxygen K-edge but also for the transitions and levels involved in the manganese L-edge. For our presented proof-of-concept X-ray spectroscopy studies of the OER, we will concentrate on the X-ray absorption and emission features of the manganese atoms in La_0.6_Sr_0.4_MnO_3_ (LSMO), i.e., the manganese L-edge transition features, since we expect their oxidation state changes during the OER. The manganese atoms are highlighted as violet points in the LSMO crystal structure shown in Figure 1A. Figure 1B depicts the resonant core excitation (XAS) and subsequent RIXS emission. For transition metals, soft X-rays promote the core 2p electrons directly into the 3d frontier states, which is dipole-allowed. The subsequent decay from the valence states carries the information crucial for understanding the material’s physiochemical properties. We yield information about the population of the electronic states of the investigated material (here a perovskite). Combining it with time-resolved *in situ* (or even *operando*) X-ray methods allows changes of the population of the electronic states to be deduced in a dynamic way. Taking into account that RIXS belongs to the core hole clock spectroscopies (Figure 1B), it is possible to gain information about the highest occupied or lowest unoccupied states of the material as well as the corresponding band gaps.

## Soft X-ray Emission Spectroscopy Developments for Synchrotrons and Free-Electron Laser Sources

By tuning the incident X-ray energy and collecting the X-ray emission integrally, or—in other words—inverting XES by monitoring particular X-ray emission spectroscopy (XES) transitions and tuning the incident X-ray energy, it is possible to collect the partial (when partially integrated) fluorescence yield (PFY) and total fluorescence yield (TFY) as functions of the incident energy. The spectra collected are comparable to the ones obtained from X-ray absorption spectroscopy in transmission mode.

However, since the X-ray photons are collected in fluorescence and scattering mode and the X-ray penetration depth sets the monitored sample thickness (Figure 1A), the sample thickness does not define the collected spectra (i.e., via saturation effects),^1^ e.g., when the PFY of the 3s to 3d transition is monitored.^2^ It is then possible to study specific selected sheets of layered compositions. An example would be layers on target materials, where the spectroscopic properties of the target materials are out of the monitoring window and thus do not contribute to the background of the collected signals. Furthermore, liquid jets can be added (as another layer), and complex reactions like the OER can be investigated. This finally leads to the possibility of utilizing the full advantages of high-flux X-ray sources (time resolution, high repetition frequency, flux, brilliance, and coherence), allowing for the development of high-resolution and multidimensional X-ray spectroscopy tools like XES or RIXS.^20−27^ This can enable more detailed investigations of the mechanisms driving the OER. Its technical realization is schematically drawn in Figure 1C, and photographs of the setup are shown in Figure 1D. At that time, we named this endstation, which was first operational in 2012 in the soft X-ray regime, the ChemRIXS endstation.^20−23^ In order to reduce background noise in the X-ray spectra, the whole experimental chamber is set to 10^–3^ mbar vacuum, which is a challenge for running the liquid jet smoothly. Radiation damage when monitoring chemical reactions with soft X-ray photons is an additional challenge. To avoid or minimize it, we have developed a vacuum-compatible liquid jet technology.

Since the liquid jet technology (Figure 1D) runs up to megahertz sample exchange rates, ChemRIXS can be coupled to high-repetition-frequency synchrotrons as well as free-electron lasers.^20−23^ ChemRIXS has been operated at PETRA-III, FLASH-I, and FLASH-II. The liquid jet is used as a megahertz sample exchange unit in the high-resolution Heisenberg RIXS apparatus at the SCS beamline of the European XFEL.^24^ The liquid jet technology can avoid typical soft X-ray radiation damage processes that allow otherwise atypical reaction pathways to occur in electrochemistry, which is especially important when studying the reactions of water splitting, namely, HER and OER.

**Figure 2:**
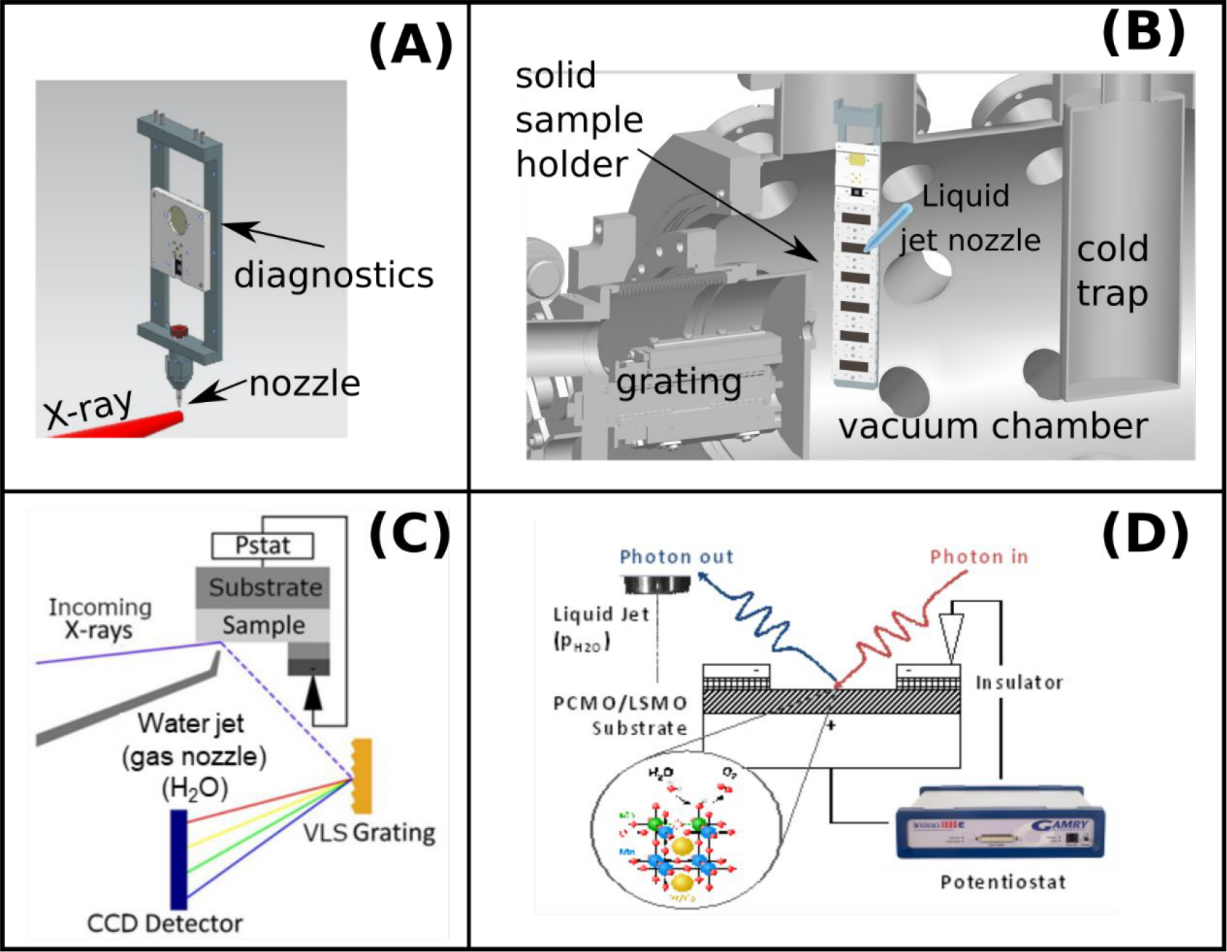
(A) Liquid jet coupling into the ChemRIXS chamber. (B) Liquid jet coupled with surface-sensitive (grazing incidence, GI) X-ray geometry for GIRIXS studies. (C) Design of the ChemRIXS-GIRIXS setup for surface/water/liquids studies, including a potentiostat for *in situ* (and in the future *operando)* GIRIXS studies. (D) Side view of the setup.

Due to the high-repetition-frequency performance of the liquid microjet and its compatibility with high-flux X-ray sources, we developed the ChemRIXS chamber for time-resolved *in situ* and *operando* studies (Figure 2).^2,20−23^ The multiuse liquid jet (Figure 2A) combines calibration tools with the liquid jet. Furthermore, it is combined with solid-state samples that are active during the OER catalysis (Figure 2B). For possible *in situ* and *operando* studies, the catalysts by themselves are coupled to a potentiostat (Figure 2C,D).^2,20−23^ For *operando* studies, an additional mass spectrometer monitors the catalytic activities. For time-resolved studies in the case of photocatalyst studies, a pulsed optical laser has been coupled in.^25−27^ Time-resolved experiments are reached when external stimuli like a pulsed optical laser are coupled in. Various proof-of-concept studies of this sort have been performed at FLASH and LCLS for XUV and soft X-ray emission spectroscopy, where we determined the electron energy and “real-time movements” (meaning investigating the population changes of electronic states of particular transition energy) in materials, thus contributing to the “film of chemical reactions” from an electron energy, electron orbital, and electron distribution point of view for molecular and homogeneous catalytic systems.

Due to the flexible geometric design of the various components, it is possible to run the RIXS spectrometers of ChemRIXS in transmission or reflection geometry or in the grazing incidence (GI) geometry as GIRIXS.^28,29^ Our time-resolved *in situ* and *operando* XES/RIXS studies can therefore be either bulk- or surface-sensitive, as shown in Figure 2C,D.

**Figure 3:**
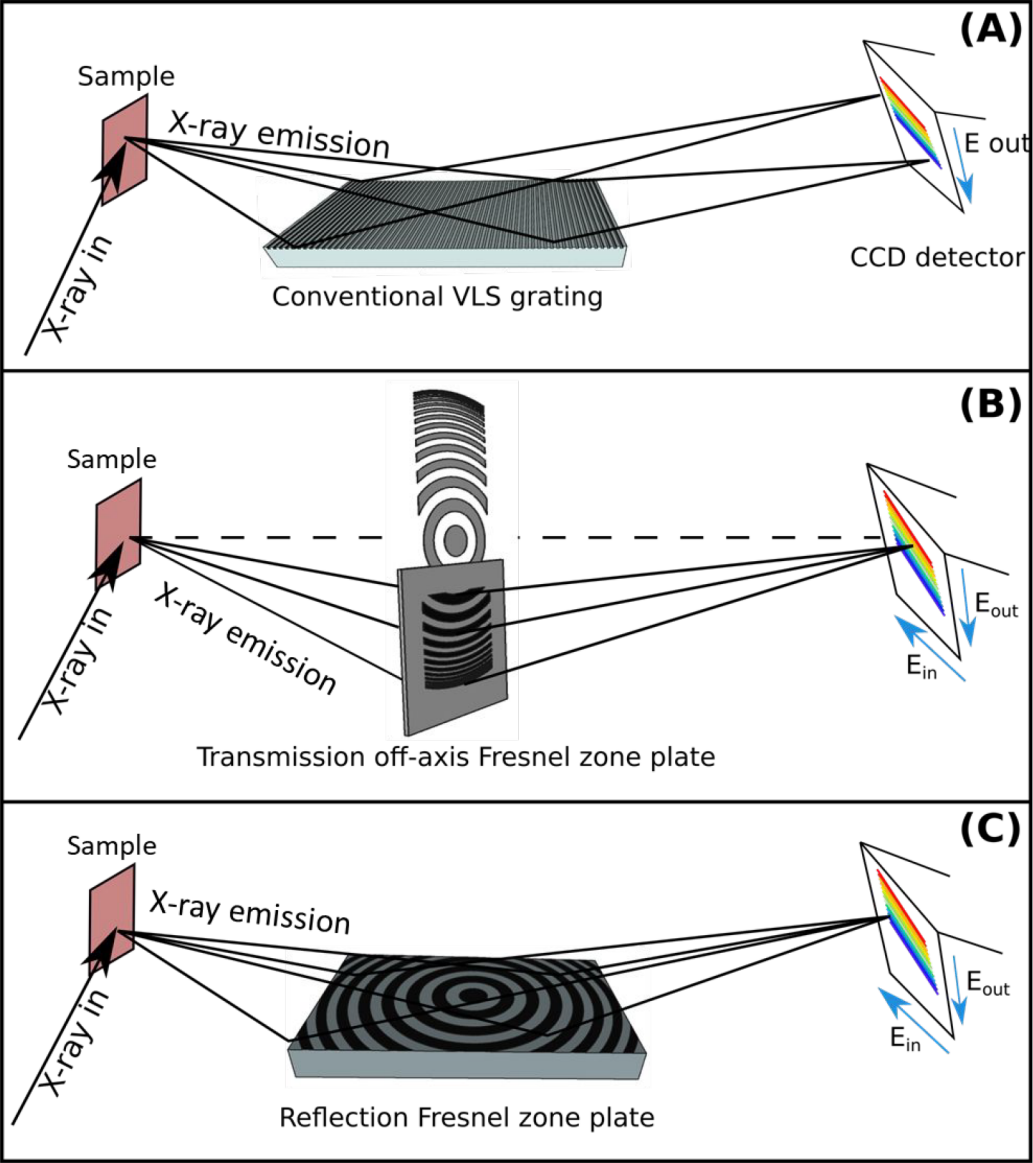
(A) Conventional RIXS detection scheme with a grating. (B) Transmission zone plate for RIXS detection. (C) Reflective zone plate for RIXS detection. All spectrometers can investigate solid-state or liquid bulks or can be operated for grazing incidence recording (GIRIXS). Utilizing the time structure of the high-flux X-ray photon sources (PETRA-III synchrotron and FLASH-I/II free-electron laser) allows for *in situ* and *operando* GIRIXS studies of surfaces with water or other liquids.

Depending on the information about the OER which we want to gather, different X-ray spectrometer types can be combined with liquid microjets in the ChemRIXS chamber.^30−34^ The exchangeable soft X-ray spectrometer units are equipped with a transmission zone plate (TZP), a reflection zone plate (RZP), and a classical grating spectrometer (Figure 3). Common for all technical approaches of soft X-ray spectroscopy, the XES and RIXS processes consist of the following steps:

- The X-ray beam hits the sample (solid/liquid beam).
- The interaction with the X-rays increases the electron energy in the material/sample under investigation in an element-specific manner.
- The sample emits photons of specific energy.
- The emitted photons hit an optical grating and are separated according to their energies.
- Photons separated according to energy hit the detector.
- A spectrum of energies can be measured via the distribution of photons on the detector.

In ChemRIXS the liquid jet can be combined with a conventional X-ray grating spectrometer, where the incident X-ray energy is wavelength-selected by a monochromator of the beamline and the outgoing X-ray photons are recorded as XES spectra as a function of incident X-ray energy (Figure 3A). In this way “RIXS maps” can be derived by a stepwise approach of tuning the incident X-ray photon energy and recording the energies of the outgoing X-ray photons scattered from the sample. This conventional way of high-resolution RIXS map collection is only efficient and realizable in a reasonable time window when high-flux X-ray sources with stable photon fluxes are coupled in. Alternatively, when energy resolution issues for the systems investigated are negligible, the recording process can technically be simplified and accelerated by combining the liquid jet with a TZP (Figure 3B) or RZP (Figure 3C), where all sample-emitted X-ray photons are locally selected according to their energies and simultaneously recorded on a 2D X-ray detector.

## *In Situ* Studies on LSMO/H_2_O/KOH Interfaces

Depending on the scientific question, with these three different types of spectrometers coupled to liquid microjets, different questions of the OER activity and the OER reaction mechanism are addressed. In the following we will present our comparative *in situ* studies of LSMO in water vapor (LSMO*H_2_O) and with alkaline solution (LSMO*H_2_O/KOH). LSMO perovskite can be used as a catalyst for either the oxygen reduction reaction (ORR) or the OER. Its perovskite crystal structure is shown in Figure 1A.

Perovskite oxides have the general structure ABO_3_, where commonly lanthanides or group II elements are found at the A sites and transition metals at the B sites.^35^ Complete or partial substitution of the A sites and/or B sites changes the physicochemical properties of the perovskite oxide, often without affecting the perovskite structure significantly. Chemical substitutions in perovskite oxides have been shown to impact their catalytic activity for the OER, making them a good choice for systematic studies.^36−39^ Highly crystalline thin films with defined orientation have been insightful model systems studied in water vapor, e.g., by ambient pressure *in situ* soft XAS,^15^*in situ* XPS/XANES,^40^ environmental TEM,^3,41−43^ or ultrafast time-resolved optical studies.^44^ Here we focus on photon-in/photon-out synchrotron spectroscopy using a liquid jet setup.

**Figure 4:**
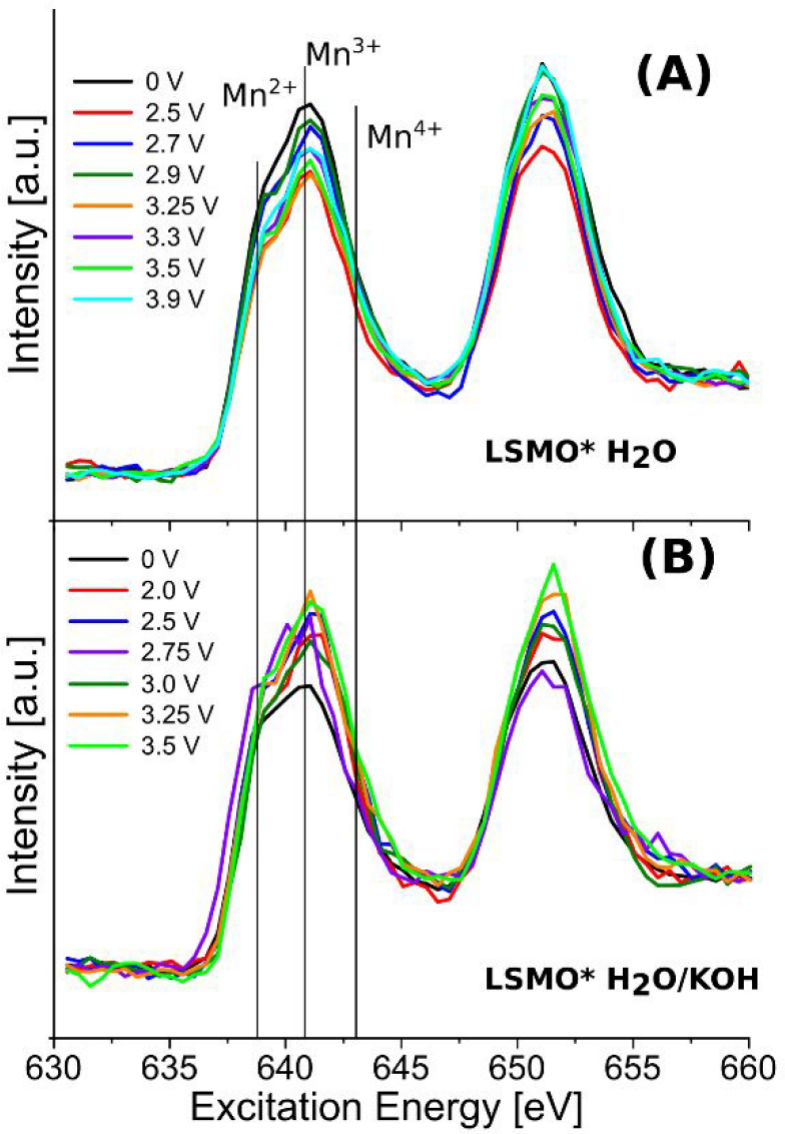
LSMO*H_2_O and LSMO*H_2_O/KOH surface reactions under voltage steps (vs ground). (A) *In situ* Mn L-edge iPFY of LSMO*H_2_O for varying voltages. (B) *In situ* Mn L-edge iPFY of LSMO*H_2_O/KOH for varying voltages. The Mn L-edge iPFYs were measured with TZP-GIRIXS spectroscopy.

**Figure 5:**
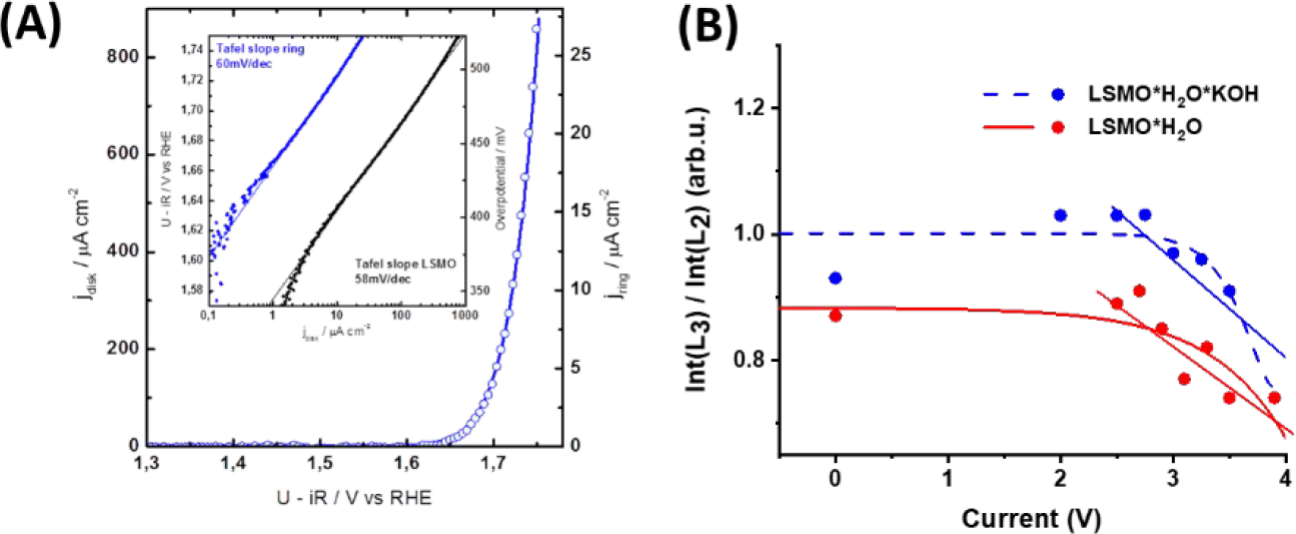
(A) Rotating ring–disk studies of the same LSMO in water system (same doping level, same synthesis approach) emphasizing a voltage threshold at around 1.75–1.8 V vs reversible hydrogen electrode (RHE). The inset shows the LSMO disk and Pt ring detecting oxygen by reduction; the big figure is an overlay of the disk and ring data. (B) iPFY intensity ratio of the Mn-L_3_ to Mn-L_2_ integral intensities as a function of applied potential. Red curve/line and red circles: LSMO*H_2_O. Blue curve/line and blue circles: LSMO*H_2_O/KOH.

**Figure 6:**
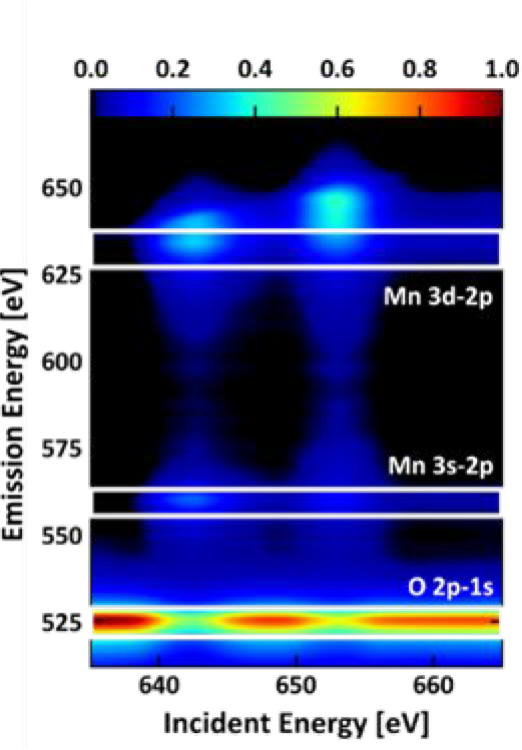
Monitoring of the LSMO*H_2_O responses and the electronic properties of LSMO*H_2_O. Utilizing the grating spectrometer, the GIRIXS maps of the Mn L-edge and O K-edge of LSMO*H_2_O are of higher resolution and allow for the investigation of electronic features beyond an elemental and oxidation state investigation.

**Figure 7:**
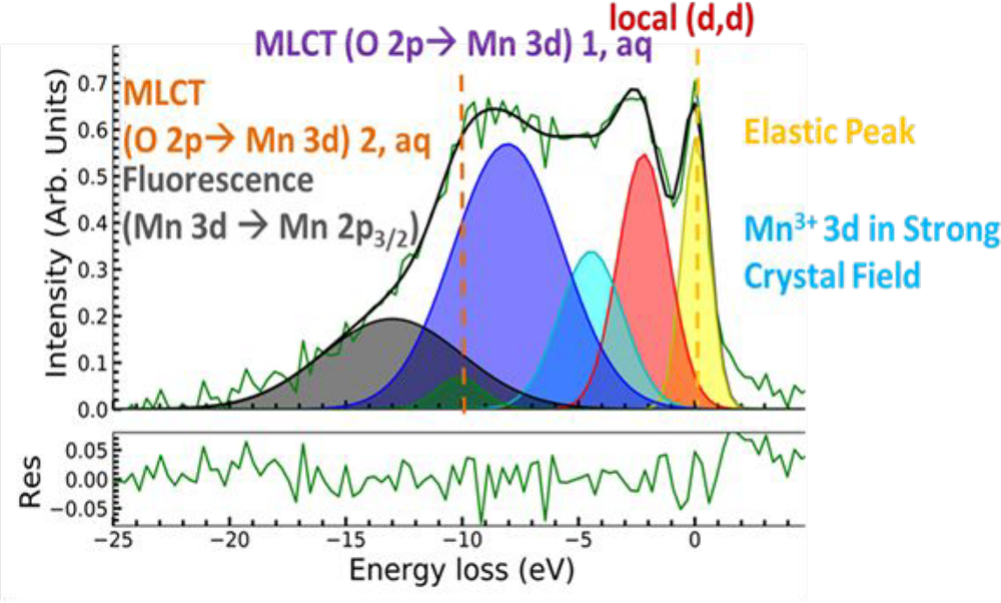
Monitoring of the LSMO*H_2_O responses and the electronic properties of LSMO*H_2_O. High-resolution XES of LSMO*H_2_O recorded with the grating spectrometer allows resolution of charge transfer features between the solvent and LSMO. The color codes are described in the text. The experiment was performed by combining the liquid jet technology with RIXS grating spectroscopy. The excitation energy for this spectrum was set to 642.8 eV.

In this work we will present the scientific results of two types of proof-of-concept OER studies on LSMO which resemble two complementary X-ray emission spectroscopy capabilities for *in situ* and *operando* studies:

(i) *A study of the OER under in situ conditions with low-resolution X-ray emission spectroscopy* (Figures 4 and 5). For the qualitative study of the electrochemistry of the bulk catalysts under applied voltage *in situ*, gaseous or liquid jets were coupled to a low-resolution transmission zone plate spectrometer. Additionally, we studied the influence of alkaline solutions on LSMO catalyst properties by analyzing the OER activity of the LSMO*H_2_O surface reaction with this low-resolution X-ray emission spectroscopy approach. The studies were also performed adding a mass spectrometer. Since during the experiments no systematic recording was performed (beyond the confirmation of oxygen production), we name the experiments *in situ* rather than *operando* according to the common definitions.
(ii) *A study of the electronic influence of the water adsorbate on the LSMO surface with high-resolution X-ray emission spectroscopy coupled to a liquid jet* (Figures 6 and 7). In the high-resolution X-ray experiment, the electronic properties of the LSMO–water surface were studied in detail by coupling the liquid jet to a grating spectrometer. This approach diminished radiation damage issues (like the creation of solvated electrons in the water layer) so that the experiment could run with a megahertz repetition rate. The increase in the rate by 3–5 orders of magnitude allows for high-resolution electronic structure studies, as shown by the X-ray emission/RIXS spectra presented in Figure 7.

In sum, (i) resembles a technical preparation study confirming that LSMO can be studied under *in situ* (or even *operando*) conditions with high-flux X-ray sources. It confirms that spectroscopic analysis deduces trends in manganese oxidation states under *in situ* conditions. The more sophisticated high-resolution *in situ* X-ray emission studies (ii), which are prepared to reduce radiation damage from the high-flux X-ray sources, can be built on the results obtained in (i) and add information about the electronic properties of LSMO when water layers are added for the *in situ* experiments.

Both types of soft X-ray spectrometry allow recording of the changes in the oxidation state or the element-specific chemical composition of the bulk electrocatalyst or water, the surface of the electrocatalyst, or the water adsorbate. In general, the highest surface sensitivity is reached during recording with the grazing incidence geometry.

### i Study of the LSMO OER under *In Situ* Conditions with Low-Resolution X-ray Emission Spectroscopy

For our *in situ* studies, we combined TZP X-ray spectroscopy with a potentiostat and monitored the Mn valence and oxidation states of LSMO with water upon voltage stepping. With the TZP-based X-ray spectroscopy, the changes in the oxidation states of various elements in LSMO can then be quickly recorded. Integrating in particular the manganese L-edge emission features (3d → 2p_1/2_ RIXS) for different applied voltages yields the manganese L-edge partial fluorescence yields as 3d-PFY. For the iPFY signals we applied the same data procedure as been described in the general procedure of integration of the RIXS features of LSMO:^2^ we obtained the iPFY of the Mn L-edge by integrating the region of interest of the O 2p–1s emission feature and inverting the signal intensity. In the following we will concentrate on the iPFY signals. Changes in (and confirmation of) catalytic activity are additionally monitored by coupling a mass spectrometer to the setup (which is not considered in this work).

On the chemical side, during the OER, water is oxidized to O_2_ by the release of electrons, which are given to the LSMO anode surface. At this particular moment of electron admission, higher-valence intermediate Mn states (Mn^3+^/Mn^4+^) are formally reduced toward lower-valence states by the admission of electrons, leading to a lower mixed or intermediate valence state (Mn^2+/3+/4+)^. Note that the occurrence of mixed or intermediate valence states of Mn can be distinguished. Intermediate valence states are present in the metallic phases of LSMO, where 3d electrons in the conduction band are delocalized. This is typically the case for valence states in between Mn^3.2+^ and Mn^3.5+^ in highly crystalline LSMO with low defect density. Mixed valence states are present in semiconducting or insulating phases of LSMO, where electric charge is rather localized and mixed valence states of Mn^2+^, Mn^3+^, and Mn^4+^ can evolve. This can be triggered by, e.g., point defects, change of doping, strain, and surface reconstruction. In Figure 4 the iPFY energies have been set through energy calibrations of reference L-edge Mn absorption spectra of defined Mn oxidation states in various manganese oxides,^15,35,44−48^ ranging over the manganese valence states of Mn^2+^, Mn^3+^, Mn^2+^/Mn^3+^, and Mn^4+^. In the current work, the beamline and part of the spectrometers have been calibrated on well-defined argon and xenon lines of soft X-ray gas detectors.^2,20^ This sets the energy of the spectrometers to 0.1–0.5 eV energy precision. The best energy resolution of the TZP, however, is about 0.8–1 eV. Independent of the initial oxidation state, applying Mosley’s law yields a +1.6 eV relative shift/oxidation state in our PFY spectra in Figure 4. With the energy resolution of 1 eV in mind, the formal calibration points are the maxima at 639.4 eV for Mn^2+^, 641.4 eV for Mn^3+^ and 643.2 eV for Mn^4+^. Additional mixed-state transitions are assigned to the transition shoulders at 639.6 eV (Mn^2+^/Mn^3+^), 640.5 eV (Mn^2+^), 642 eV (Mn^2+^/Mn^3+^), and 643.8 eV (Mn^4+^), also for mixed valences like in Mn_3_O_4._^46^ The assignments of the respective calibration points are marked as vertical lines in the PFY spectra in Figure 4.

The present OER studies of LSMO (as the anode) under more realistic oxidation conditions reveal also a more precise monitoring of the dynamic LSMO oxidation state changes (Figure 4) and suggest that a more complex manganese oxidation state mixture is involved in the LSMO-catalyzed OER. Figure 4 also emphasizes that we already have intermediate/mixed valence states reaching from Mn^2+^ to Mn^4+^ in LSMO before the OER and also on the surface. Since also Mn^2+^/Mn^3+^ mixed valence transitions are found at around 639.4 eV and the reference is the zero voltage (vs ground) LSMO form, we interpret the existence of the shoulder and its variation during voltage sweep or surface coverage as a change in the manganese valence state toward smaller values, i.e., from Mn^3+/4+^ to Mn^3+^ or even mixed Mn^2+/3+^ states, etc. It is safe to state that activating LSMO by increasing the applied voltage leads also to a slight reduction of the surface Mn valence states in LSMO, which are not fully recovered by the charge flow back from the bulk and which we will use for the following interpretations as a type of intrinsic LSMO anode feature without water adsorbate and electrolyte around.

Figure 4A shows the *in situ* inverted partial fluorescence yield (iPFY) of LSMO*H_2_O, and Figure 4B presents the *in situ* studies of the iPFY of LSMO*H_2_O/KOH when the water vapor in the chamber forms a KOH/water mixture with a KOH-covered LSMO film and under voltage sweep. In Figure 4A, the intensity of the iPFY of LSMO*H_2_O decreases the first 639 to 643 eV iPFY transition band when the voltage is increased from 0 to 3.9 V, and the second iPFY transition band with its maximum at 652.5 eV increases.

To increase the chemical parameter space of systematic analysis of the OER activity of the electrocatalyst, it is important that the RIXS setup includes additional (chemical experimental) degrees of freedom. Therefore, the studies have been extended from water to alkaline solution covering LSMO. Alkaline solution can be created by either adding water to the salt covering the LSMO surface or using mixing units precoupled to the nozzle and transfer tube for water in a microjet mixing unit that can mix different solution channels, as described previously^50,51^ and shown in Figure 1B. When alkaline solution is added to the LSMO surface, at 0 V vs ground the PFY looks quite similar to the ones of LSMO covered with water without applied voltage potential (Figure 4A). Also here most prominent are the increase of the low-energy transition shoulder of the *in situ* PFY when the voltage is ramped up. Within the resolution of the recorded data, the found behavior is quite similar to that of LSMO covered with water (Figure 4A), suggesting a reduction mechanism similar to that discussed in the case of LSMO*H_2_O: when voltage potential is applied, after a specific threshold, again a 639.4 eV transition shoulder appears in the iPFY due to the formation of lower Mn^2+^/Mn^3+^ mixed valence states.

The current spectrometers are not sensitive to eventually fully dissolved Mn ions in water or in alkaline solution (with KOH). The phase evolution at different pH and during oxidation has been previously reported for the Mn-based OER catalysts, and they are found to exhibit high structure flexibility that relates to their OER behavior.^52^

Formally, the valence state change of Mn can be monitored by calculating the iPFY intensity ratio of the Mn-L_3_ to Mn-L_2_ integral intensities assuming that the Mn-L_3_ edge height is reduced and that other ratios are thus expected for undistorted data. This method has successfully been developed for *in situ* electron energy loss spectroscopy of LSMO for getting a deeper insight into *in situ* studies.^40,41^ Since our PFY spectra are quite distorted (the 3d-PFY even more than the iPFY presented in this work), this analysis can only be seen as a first zeroth-order approach emphasizing the proof of concept of the present study. Figure 5 shows the iPFY intensity ratio of the Mn-L_3_ to Mn-L_2_ integral intensities as a function of applied potential. The iPFY ratio (Figure 5B) is compared to rotating ring–disk studies on the same system (Figure 5A).^49^Figure 5B compares the intensity ratios between the L_2_ and L_3_ edges for LSMO covered with water (LSMO*H_2_O) and LSMO covered with alkaline solution (LSMO*H_2_O/KOH). For LSMO*H_2_O (red curve) and LSMO*H_2_O/KOH (blue curve), a clear tendency to a thresholdlike behavior has been found.

In Figure 5B, the ratio onset of LSMO*H_2_O is set to 1 for no voltage applied, and that of LSMO*H_2_O/KOH is slightly smaller (0.8). The found voltage-independent abscissa offset decreases in the integral intensity ratio of LSMO*H_2_O and LSMO*H_2_O/KOH can be explained by the decrease of the emitted photon signal due to the added water/alkaline solution layer and the energy-dependent absorption of the added water/alkaline water layer to LSMO.

For both, LSMO*H_2_O and LSMO*H_2_O/KOH, a voltage threshold of >2.2 V is found, from where the ratio then monotonically decreases as the voltage increases from 2.2 to 4 V vs ground. We hypothesize that the reduction of Mn is due to water/hydroxide oxidation and thus correlate the onset of this reaction with the onset of the OER on LSMO in rotating ring–disk electrode (RRDE) laboratory experiments at 1.65–1.7 V vs RHE, shown in Figure 5A.^49^ The RRDE experiments were performed on the same LSMO system, synthesized in the same way and with the same doping level. The deviations between our found *in situ* studies and the ones from the laboratories can be explained in the following way: first, our proof-of-concept studies presented here are technically not as much optimized as the ring electrode experiments, as already the statistics of both experiments suggest. Second, our applied voltages are not electronically experimentally referenced in the same way as the ring electrode experiments. Third, the ratio method utilizing TZP spectroscopy misses some sensitivity (distorted PFY spectra, etc.) which adds an insensitivity on the ratio scaling (so the ratio changes are detected at higher values than the original ones). Fourth, mechanistic differences may also influence the voltage threshold value since the LSMO sample environment slightly differs in comparison with a ring electrode experiment and the LSMO water layers produced in this experiment. Overall, Figure 5B emphasizes that it is possible with the explained experiment to monitor *in situ* LSMO activity. However, it also emphasizes that more detailed electronic and electronic–structural studies are needed to understand completely the LSMO water coverage.

### ii Study of the Electronic Influence of the Water Adsorbate on the LSMO Surface with High-Resolution X-ray Emission Spectroscopy

When the liquid jet/LSMO is coupled to our high-resolution X-ray emission spectrometer, we can monitor in detail with greater sensitivity the formation of water layers adsorbed on LSMO. From the TZP-based PFY studies explained in the last paragraph, we know that when water is added to the surface but no voltage potential is applied, the valence states of surface Mn are not reduced to pure Mn^2+^ or Mn^3+^ or intermediate Mn^2+^/Mn^3+^ valence states. The discussed inconsistencies and normalization furthermore suggest that there may be more modifications in particular on the LSMO surface that we have not taken into account yet.

With grating-based X-ray spectroscopy and utilizing the megahertz repetition frequency of the synchrotron, RIXS maps of the system can be recorded in a reasonable time. Utilizing the liquid jet reduces the probability of radiation damage. Consequently one RIXS map with the X-ray emission features versus the incident X-ray energy (Figure 6) can be derived which gives a spectral overview over the oxygen K-edge features and the manganese L-edge features (Figure 6) in one map. In the grating experiment, the X-ray transitions between the oxygen K-edge of bulk water and surface water and the ones of the perovskite sublattice in LSMO are well-separated, allowing the partial fluorescence yields of the different oxygen species present in the OER and under the experimental conditions to be distinguished.

Figure 7 presents the high-resolution XES spectrum of the manganese L-edge of LSMO*H_2_O recorded with a grating spectrometer. The XES spectrum can directly be energy-calibrated to the elastic peak (yellow line). Additional different mixed/intermediate Mn^3+^/Mn^4+^ valence states can be assigned: the resolved transition bands belong to the local d–d transition (orange transition bands), the Mn 3d → 2p_1/2_ fluorescence (gray transition bands), and some Mn^3+^/Mn^4+^ emission bands in the strong crystal field (cyan transition bands). These transitions have also been found in other LSMO XES studies.^47,48^ Upon addition of water to LSMO, in the XES spectrum of Figure 7 particularly for the LSMO*H_2_O water adsorbate additional transition bands have been observed that were not recorded in our earlier studies of dry LSMO *in vacuo*.^2^ We assign these additional O 2p→ Mn 2p emission transition bands to charge transfer from the oxygen atom of the adsorbed water to the manganese atoms of the LSMO surface, abbreviated as MLCT_aq_ and highlighted with orange and purple transition bands in Figure 7. These MLCT_aq_ states alter the activation properties of the surface Mn sites in LSMO even before application of a potential.

## Conclusion

We can conclude that the current advances of liquid jet technology combined with state-of-the-art spectroscopy at high-flux X-ray sources allow the study of the electronic, structural/electronic, and mechanistic properties of metastable states as well as very short-lived intermediates. Liquid jet technology not only contributes to time-resolved studies but can also enhance *in situ* (and *operando*) studies since it reduces the probability of accumulating radiation damage. Depending on the X-ray spectrometer added, qualitative or quantitative chemical analysis, like oxidation state changes or element-specific composition changes, can be monitored *in situ*. Due to additional mixing capabilities, liquid jet technologies add further degrees of freedom for the chemical composition. For an in-depth mechanistic study, however, high-resolution X-ray spectroscopy is of advantage. For studying the water-splitting OER electrocatalytic reaction of LSMO perovskite, high-resolution grating studies allow monitoring of transient charge transfer states, the filling of empty LUMOs of LSMO through the adsorption of water, etc. However, all of these novel experimental approaches are only possible through the versatile developments of liquid jets and liquid beam technologies.
